# Transcriptional regulation prediction of antiestrogen resistance in breast cancer based on RNA polymerase II binding data

**DOI:** 10.1186/1471-2105-15-S2-S10

**Published:** 2014-01-24

**Authors:** Denan Zhang, Guohua Wang, Yadong Wang

**Affiliations:** 1 Center for Bioinformatics, School of Computer Science and Technology, Harbin Institute of Technology, Harbin, 150001, China

**Keywords:** transcriptional regulation, antiestrogen resistance, prediction of TFs, RNA polymerase II

## Abstract

**Background:**

Although endocrine therapy impedes estrogen-ER signaling pathway and thus reduces breast cancer mortality, patients remain at continued risk of relapse after tamoxifen or other endocrine therapies. Understanding the mechanisms of endocrine resistance, particularly the role of transcriptional regulation is very important and necessary.

**Methods:**

We propose a two-step workflow based on linear model to investigate the significant differences between MCF7 and OHT cells stimulated by 17β-estradiol (E2) respect to regulatory transcription factors (TFs) and their interactions. We additionally compared predicted regulatory TFs based on RNA polymerase II (PolII) binding quantity data and gene expression data, which were taken from MCF7/MCF7+E2 and OHT/OHT+E2 cell lines following the same analysis workflow. Enrichment analysis concerning diseases and cell functions and regulatory pattern analysis of different motifs of the same TF also were performed.

**Results:**

The results showed PolII data could provide more information and predict more recognizably important regulatory TFs. Large differences in TF regulatory mode were found between two cell lines. Through verified through GO annotation, enrichment analysis and related literature regarding these TFs, we found some regulatory TFs such as AP-1, C/EBP, FoxA1, GATA1, Oct-1 and NF-κB, maintained OHT cells through molecular interactions or signaling pathways that were different from the surviving MCF7 cells. From TF regulatory interaction network, we identified E2F, E2F-1 and AP-2 as hub-TFs in MCF7 cells; whereas, in addition to E2F and E2F-1, we identified C/EBP and Oct-1 as hub-TFs in OHT cells. Notably, we found the regulatory patterns of different motifs of the same TF were very different from one another sometimes.

**Conclusions:**

We inferred some regulatory TFs, such as AP-1 and NF-κB, cooperated with ER through both genomic action and non-genomic action. The TFs that were involved in both protein-protein interactions and signaling pathways could be one of the key resistant mechanisms of endocrine therapy and thus also could be new treatment targets for endocrine resistance. Our flexible workflow could be integrated into an existing analytical framework and guide biologists to further determine underlying mechanisms in human diseases.

## Background

Breast cancer is not only one of the most common cancer but also the most fatal cancers in women worldwide [1]. Previous studies showed that estrogen signal plays a critical role in pathogenesis and development of breast cancer [2-5]. For same reason, endocrine therapy impeding estrogen-ER signal reduces breast cancer mortality and becomes a mainstay of breast cancer treatment. Endocrine therapy counteracts the effect of estrogen by either reducing the source of estrogen or blocking the estrogen signaling pathway in breast cancer cells without significant effects on normal cells. Since 1973, antiestrogen preparations of tamoxifen (TAM) have been widely used in endocrine therapy for breast cancer, and TAM is considered the standard treatment for estrogen receptor (ER)-positive patients until now. Unfortunately, approximately 40% of patients relapse after endocrine therapies [3]. Therefore, an essential understanding of the biological mechanism of antiestrogen resistance, especially the comprehension of TFs (transcription factors) regulation differences at transcriptional regulation level, will greatly promote the development of new drug targets discovery or novel treatment methods for breast cancer.

Many previous studies about the regulatory abilities of TFs based on gene expression data. Conlon et al. discovered sequence motifs on upstream of genes, especially expression-mediating motifs of medium to long length with multiple degenerate positions that undergo expression changes in a given condition by combining the advantages of matrix-based motif finding and oligomer motif-expression regression analysis [6]. A cross-gene identification scheme was proposed by Lin et al. to infer how multiple TFs coordinate to regulate gene transcription in the yeast cell cycle and to uncover hidden regulatory functions of a cis-regulatory circuit based on the dynamic model of cis-regulatory circuits and microarray data [7]. Based on the statistical analysis of TF binding through microarray and TF-DNA interaction data, Ryu et al. identified regulatory modules that include all combinations of TFs, plus a number of binding constraints in target genes [8]. He et al. presented a method focused on 'active' TFs that regulate the real-time expression of genes [9]; they used an enhanced Bayesian classifier to predict pairs of TFs and target genes based on time-course expression data. Recently, Ahmed et al. considered the impact of CNV (copy number variation) in gene expression. They built a linear model to depict the regulation between regulatory TFs, CNVs in each cell line and genes that were differentially expressed in 305 human cancer cell lines [10]. Geeven et al. proposed an approach that was also based on linear model to predict TF-gene expression associations and TF-TF interactions from experimental data. Their approach was a four-stage method based on lasso and post hoc re-sampling to identify and prioritize synergistic interactions between predictors that underlie observed variations in gene expression [11]. To gene expression, the transcription process is regulated by TFs and the post-transcriptional modification process is regulated by microRNA. Although these studies attempted to improve their predictions' accuracy regarding transcriptional regulation by a variety of methods based on gene expression data, they have not yet succeeded in eliminating the essential impact of post-transcriptional modification.

From a macro point of view, ER function can be divided into two modes of action during breast cancer initiation and progression. The genomic action mode mediates genomic transcription regulation through nuclear-initiated steroid signaling, and the non-genomic mode activates various protein kinase cascades [4]. Both the genomic and non-genomic pathways play a role in the response to estrogen signaling and are regulated by TFs in breast cancer cells. Furthermore, the genomic and non-genomic mechanisms of action of the ER are not mutually exclusive, but many interactions exist between these two modes [4]. Previous studies about endocrine resistance in breast cancer have shown that TAM-resistant sublines (OHT) often show changes at the level of transcriptional regulation compared with wild-type breast cancer (MCF7) cells [12]. Therefore, we are more concerned about the role of the regulation of transcription levels of breast cancer cells with antiestrogen resistance, and we believe that a better understanding of the mechanisms of acquired resistance to endocrine therapy could point to novel strategies or new therapeutic targets that could facilitate further improvements in breast cancer treatment.

ER is a key protein implicating in the majority of breast cancers. Also many previous studies based on ER immunoprecipitation data. However, only focusing on the ER could ignore those alternative regulatory mechanisms in breast cancer, especially in estrogen-independent breast cancer. In addition, gene expression is influenced by post-transcriptional regulation, which will reduce the accuracy of the predicted transcriptional regulation. In cells PolII drives the vast majority of the transcription process and is widely recognized as part of the general transcriptional machinery. Because there is a wide range of representative and quantitative PolII binding in an individual gene, the amount of PolII binding will be more accurate than the amount of gene expression as a measure of transcriptional regulation. For these reasons, we attempted to further reveals the TFs involved in antiestrogen resistance to breast cancer and their cooperative relationships by analyzing PolII immunoprecipitation data.

We sought to find a different regulatory mode of TFs by comparing E2-stimulated samples with control samples as references and revealing biological characteristics that differ between the MCF7 and OHT breast cancer cell lines. For this reason, we proposed a two-step workflow to investigate the relationships between the TFs involved in regulation based on the PolII binding quantity in the gene promoter region. To ensure comparability between the samples, we first normalized the PolII binding quantity in both the MCF7 and OHT cell lines before and after E2 stimulation [13,14]. For genes with differential PolII binding quantity under E2 stimulation, we attempted to identify possible regulatory TFs in their promoter regions. Finally, according to the results obtained by the stepwise regression model, we predicted significant regulatory TFs of MCF7 and OHT cells by E2 stimulation.

Many previous researches predicted TFs in the regulation of antiestrogen resistance in breast cancer based on gene expression data. Here, we compared the prediction results of gene expression data and PolII binding quantity data following the same workflow. Compared with previous literature, we showed that PolII data could predict regulatory TFs with higher sensitivity and specificity. In addition, we further inspected the interaction between certain TFs and discuss the role that these key regulatory TFs play in alternative pathways of estrogen signaling.

Through comparative analysis of these results, we showed that PolII data provide richer and more accurate information compared with expression data for the prediction of regulatory TFs in breast cancer cells. In addition, the analysis workflow presented here can be very easily integrated into other existing analytical frameworks or applied to the processing of other types of data because of its simplicity and flexibility.

## Methods

Because hormonal exposure is regarded as the best characterized risk factor for breast cancer, MCF7 (17β-inducible breast cancer cell line) and OHT (TAM-resistant subline of MCF7) were chosen to build model. We hoped to establish a model of differential regulation by TFs by comparing the treatment samples (i.e., MCF7+E2 and OHT+E2) with the corresponding control samples (which were used as a reference) and uncovering biological characteristics that differed between wild-type and TAM-resistant breast cancer cell lines. PolII plays an essential role in gene transcription, including roles in recruitment, initiation, elongation and dissociation [13,14]. Thus, we used PolII ChIP-seq data from four different experiments in breast cancer cells to detect regulation by TFs at the transcriptional level in both the MCF7 and OHT cell line. In this section, we provide brief descriptions of the data used; the normalization method, which was followed by the LOWESS (locally weighted scatterplot smoothing) technique; the π-value [15] measure, which was used to filter genes with differential PolII binding quantities in their promoter regions; and a two-step workflow for the prediction of regulatory TFs.

### Breast cancer data

We applied our algorithm to PolII ChIP-seq data generated by Feng and Liu [16] in MCF7 human breast cancer cells (American Type Culture Collection, Manassas, VA, USA) and OHT before and after treatment with E2. Chromatin immunoprecipitation (ChIP) for PolII (sc-899 × and sc-8005 X, Santa Cruz, CA) was performed as previously described [17]. ChIP libraries for sequencing were prepared following standard protocols from Illumina (San Diego, CA), as described in [16]. Image analysis and base calling were performed with the standard Illumina pipeline, and the samples were run in duplicate. All of the data used here can be downloaded from http://compbio.iupui.edu/group/6/pages/mirpromoter.

### Normalization

To compare across multiple samples, we implemented LOWESS [18] normalization method to correct the mean of the observed data. Previous studies normalized data by using the total number of mapping reads (TMRs) in each sample or the sequencing depth [19-22]. This straightforward normalization scales the raw data by a constant factor and is prone to bias caused by unequal variance in different genomic regions. Compared with the TMR, our LOWESS normalization method was better equipped to remove bias and systematic errors.

Our LOWESS normalization bases on the idea of the  versus  plot, where  is the difference in log PolII binding quantity and  is the average of log PolII binding quantity. Let n refer to total number of bins in a chromosome, and j=1,2 refers to control (reference) and treatment samples, $x_{ij}$ is the PolII binding quantity for bin i(i=1,…,n). To balance the number of data points and the resolution we choose 1kbp as bin size in our application. Thus, $x_{ij}$ is the sum of the fragment counts mapped between location (i-1)×1000+1 and i×1000 in sample . Then, we can calculate $M_{i}$ and $A_{i}$ as follows:

$$M_{i}={\text{log}}_{2}\left(\frac{x_{i1}}{x_{i2}}\right)$$

$$A_{i}={\text{log}}_{2}\left(x_{i1}\times x_{i2}\right).$$

A normalization curve is fitted to this  versus  plot using LOWESS. LOWESS is a regression modeling method that combines multiple regression models in a k-nearest-neighbor-based meta-model [18]. The fits based on the normalization curve are $\hat{M}$ thus normalization adjustment is $M_{i}^{\prime }=M_{i}-\hat{M}$. The adjusted PolII binding quantities in each bin are given by the following formulas:

$$x_{i1}^{\prime }=2^{A_{i}+\frac{{M^{\prime }}_{i}}{2}}$$

And

$$x_{i2}^{\prime }=2^{A_{i}-\frac{{M^{\prime }}_{i}}{2}}$$

After the PolII read quantities in each bin were corrected, we obtained every read normalization weight $r^{\prime }$through $r_{i1}^{\prime }={x^{\prime }}_{i1}/)n_{i}$ and $r_{i2}^{\prime }={x^{\prime }}_{i2}/)n_{i}$, where $n_{i}$ refers to the PolII read counts in every bin. Based on the normalized weight of these reads, we were able to analyse the characteristics of a specific area of MCF7 and OHT genome.

### Identification of differential PolII binding quantity genes by π-value measures

Genes that showed a systematic difference between two conditions were considered to be differentially expressed [23]. Identification of differential genes through p-value or fold change measures would be some problems. When genes' expression is small, a slight expression variance can result in a significant p-value. However, a small expression change has questionable biological justification, which frequently leads to a false discovery. In contrast, some dysregulated genes in a disease condition with considerable fold change could possess a large variance but accompany a non-significant p-value. Thus makes it possible to miss these biologically meaningful changes. To avoid above problems, YF Xiao et al. [15] proposed a gene significance score called π-value, which combines the fold change and p-value into one score for the robust selection of differentially expressed genes. However, many previous studies use gene expression levels to estimate the amount of cellular transcription. In fact, the amount of gene expression also includes the influence of post-transcriptional regulation, and thus, the PolII binding quantity in cells is more accurate than gene expression and more directly reflects the transcription process. We used the π-value measure to identify genes for which the PolII binding quantity of the promoter region underwent tremendous change before and after E2 treatment.

$p{\left(k\right)}_{i}$ is the PolII binding quantity of the i-th gene's promoter in the sample class $S_{k}$ (k=1,2 represents the control and E2 the treatment); then, the log-ratio and log-fold change of this PolII binding quantity are denoted as $x_{i}=p{\left(1\right)}_{i}-p{\left(2\right)}_{i}$ and $\varphi_{i}=|p{\left(1\right)}_{i}-p{\left(2\right)}_{i}|$, respectively. Given $\varphi_{i}$ and $P_{i}$, we defined the π-value as:

$$\pi_{i}=\varphi_{i}\cdot \left(-{\text{log}}_{10}P_{i}\right)$$

where $\varphi_{i}$ is the log-fold change and $P_{i}$ is the p-value of PolII binding quantity in the i-th gene's promoter region, which results from a Fisher's exact test. The π-value is non-negative; greater π-value indicates more significant changes in PolII binding quantity. We chose a mean value that adds three times the variance of all π-values as a threshold to obtain genes for which PolII binding quantity is visibly changed by E2 treatment. These chosen genes were used as a typical sample set, and transcriptional regulation of these genes was investigated under different biological conditions.

### Identification of the potential TFBS in promoter region

The binding of TFs in promoter region is one of essential processes for transcriptional regulation in cells. To preliminarily determine which TFBSs (transcription factor binding sites) were present in each gene's promoter region, a sequence match scoring was performed on sequences of these regions in MCF7 and OHT cells. The position weight matrices (PWMs) for scoring sequence matching were obtained from the TRANSFAC and JASPAR databases, including 460 experimental confirmations of human TFs. In each gene's promoter sequence (-600 bp ~ +500 bp around the transcription start site or TSS), the PWM scored sequence by a sliding window. After scoring for each TF of the PWMs of all sites on 34,055 gene promoter regions, we chose the 2,000th-highest score as a threshold value and regarded the locations with scores above this threshold as the potential TFBSs for next prediction. With this step, we obtained a potential binding site matrix  that contains 460 human TFBSs and 34,055 promoter regions. Each row of the matrix represents a gene, and each column represents a TF; if the j-th TF binding site is present in the promoter region of the i-th gene, then the corresponding value $R_{ij}$ is its PWM score. Otherwise, the score is 0.

### Workflow to detect regulatory TFs in MCF7 and OHT cells

We propose a two-step workflow to predict active TFs in gene regulation before and after E2 stimulation in MCF7 and OHT cells. First, we constructed a linear model to describe the relationship between genes with a significant PolII binding quantity change in promoter regions before and after E2 stimulation and the TFs binding these regions, represented by TFBSs, as shown in the equation below:

$$P=R_{i}X_{i}+b.$$

where  is the PolII binding level change rate, $\text{log}\left(\frac{SK_{Treatment}}{SK_{Control}}\right)$, e.g., the logarithmic ratio of the amount of gene expression or PolII binding reads for the SK gene in the treatment and control conditions; $R_{i}$ is the previously obtained matrix of potential binding positions in gene promoter regions; $X_{i}$ reflects the regulatory ability of the TF; and  is an error term. $X_{i}$ could be estimated using the least squares method. Here, we iterated 1,000,000 times to perform the calculation, as in our previous work [24]. Each time, we randomly selected 5 of the 460 TFs to fit with the PolII binding level change rate P. And the error was estimated using the following formula:

$$E=\overset{n}{\underset{k=1}{\sum }}{\left\{p_{k}-\underset{i\in R}{\sum }R_{i}{\hat{x}}_{i}\right\}}^{2}.$$

Here ${\hat{x}}_{i}$ is the predicted regulatory ability of $TF_{i}$ and E represents the estimated error. Because a smaller model error implies a more influential binding site, a TF regulatory capacity (RC) was assigned to each selected candidate in the set according to the following formulation:

$$RC_{i}=\underset{c\in C}{\sum }\frac{1}{E_{c}^{a}}.$$

Here E is the model error, c is a subset of all of the regulatory sequences that contain the i-th TFBS and α is the power factor that influences the effect of single selections. A larger α value usually amplifies the additive contribution of the motif sets with smaller model errors in each iteration, and we set α = 5. We used the top 10% according to the RC score (in descending order) in the MCF7 and OHT cells as candidate regulatory TFs in E2 stimulus condition. Because the top 10% is an arbitrary threshold, we must further filter out the real regulatory TFs.

In the second step, we used stepwise regression method to filter out the insignificant TFs in gene regulation processes. The candidate top 10% TFs at the differentially bound promoter regions was used by the stepwise regression procedure to fit a multiple regression model:

$$P=\overset{M}{\underset{m=1}{\sum }}R_{m}S_{m}+\varepsilon .$$

Here  is log of the PolII binding level change rate of the gene, $S_{m}$ is regression coefficient and  is gene-specific error term. Initially, stepwise regression considered all of prospective TFs in fitting and tested the deletion of each variable using a chosen model comparison criterion at each step. If deleting any of TFs would significantly improve the model, then the TF was deleted, and the process was repeated. The final model was reached when no TF with a significant coefficient could be removed.

To improve the sensitivity of prediction, we performed this workflow after removing the promoter intervals with fewer than five PolII binding reads. Simultaneously, to exclude the influence of specific chromatin structure, we based our predictions on a series of sub-intervals and eventually merged those sub-predictions to obtain final result. These gene intervals were from -400 bp ~ +200 bp to -600 bp ~ +500 bp (5'UTR range) around TSS, expanding both ends by 100 bp. Then, we determined the gene set on each gene interval by differential PolII binding quantity and executed the workflow to a two-step prediction; finally, P <0.01 was considered significant for the finalized regulatory TFs.

### Inference of the TF regulatory interaction network in MCF7 and OHT cell lines

To further compare the differences in TF regulatory mode between MCF7 and OHT cell lines and provide insight into roles of different regulatory factors and their interactions in TAM resistance of breast cancer, we constructed TF regulatory interaction networks based on the significant interaction score (SIS) between TFs in MCF7 and OHT. The regulatory interaction between every pair of previously predicted TFs in each cell line was considered to build this TF regulatory interaction network. We proposed the SIS as a measure to compare the significance of the interaction between two TFs, shown as follows:

$$SIS_{ij}=-\text{log}\left(P_{ij}\right)\times \left(\frac{Int_{ij}}{Int_{i\cdot }}+\frac{Int_{ij}}{Int_{j\cdot }}\right).$$

Where $P_{ij}$ represents P-value of interactions between $TF_{i}$ and $TF_{j}$ coming from Fisher's Exact Test. $Int_{ij}$ is the number of interactions between $TF_{i}$ and $TF_{j}$, $Int_{i\cdot }$ and $Int_{j\cdot }$ indicate all of interactions that $TF_{i}$ and $TF_{j}$ participated in, respectively. Accounting for the possible differences in the level of TF interactions between different populations, we regarded the mean + variance of the SIS score as a significant threshold to infer the TF regulatory interaction network.

## Results

### Demonstration of the LOWESS MA normalization

**Figure 1:**
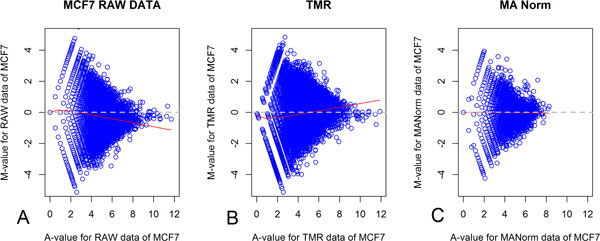
**Comparison of raw data, TMR and LOWESS MA normalization of PolII data**. We applied different normalizations to PolII binding quantity data from all chromosomes of MCF7 cell line. Chr5 is used here as an example. (A) Raw data with a clear bias toward the negative direction; (B) data normalized through the TMR method, with a clear bias toward the positive direction; (C) data normalized through the LOWESS MA method with respect to a mean of 0. Red lines represent the LOWESS smoother line with respect to the mean, and grey (dotted) lines represent the zero-difference line.

In this article, data normalized using the LOWESS MA method, which considered both global and local distribution characteristics, were used to minimize as much as possible the impact of bias and systematic error that arises from the data generation process, thus maintaining the characteristics of original data. Here, we demonstrated the effect of comparing raw data, the TMR normalization method and the LOWESS MA normalization method described above on the normalization processing of the PolII binding quantities between MCF7 cells and E2-treated MCF7 cells. We show Chr5 as an example, but results were similar across all chromosomes. Figure 1 shows a comparison between the raw data, TMR normalized data and our LOWESS MA normalized data as an example. It is apparent from the figure that the unnormalized raw data are clearly biased toward the negative direction. After TMR normalization, the data become clearly biased toward the positive direction. This result indicates that corrections based on the global data distribution characteristics, such as TMR, often cause large deviations in the area of biological functions. Considering both global and local distribution characteristics, data are normalized through the LOWESS MA method with respect to a mean of 0 and are thus normalized in the best manner.

### PolII binding data provide more regulatory information than gene expression data

**Figure 2:**
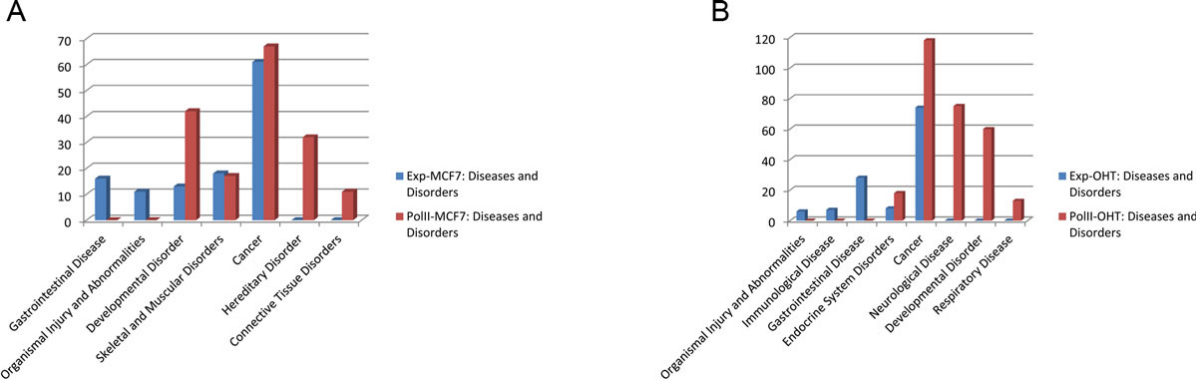
**Comparison of enrichment analyses for diseases and disorders**. Differentially regulated gene sets analyzed in MCF7 and OHT cells were obtained from PolII data and gene expression data, respectively. (A) Comparison of molecules enriched in various categories of diseases and disorders based on PolII and gene expression data in MCF7 cells. (B) Comparison of molecules enriched in various categories of diseases and disorders based on PolII and gene expression data in OHT cells.

**Figure 3:**
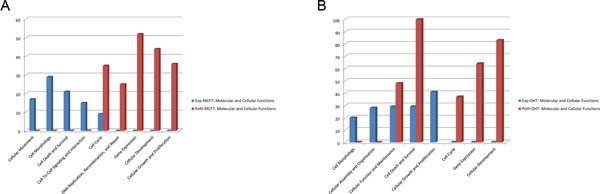
**Comparison of enrichment analyses of molecular and cellular functions**. Differentially regulated gene sets analysed in MCF7 and OHT obtained from PolII data and gene expression data respectively. (A) Comparison of molecules enriched in categories of molecular and cellular functions based on PolII and gene expression data in MCF7. (B) Comparison of molecules enriched in categories of molecular and cellular functions based on PolII and gene expression data in OHT.

Many previous studies about the regulatory mechanism differences between estrogen-dependent and estrogen-independent breast cancer based on gene expression data [25,26]. PolII has been recognized as a component of the general transcriptional machinery and drives the transcription process in cells. Because of its broad representation and metrizability, we used the PolII binding quantity as a measure of the genes' transcriptional activity. We compared the prediction results of regulatory TFs based on PolII binding quantity data and gene expression data taken from MCF7/MCF7+E2 and OHT/OHT+E2 cell lines following the same analysis workflow. We found some differences between filtered sets of differentially regulated genes obtained from gene expression data and PolII binding quantity data. For selection of differentially regulated genes, we regarded genes with π-value score greater than mean +3 variances (taken over all of the genes) as significantly differentially regulated genes. Notably, we believe the new significance indicator based on the mean and variance can reduce the impact of the different distributions of the samples and make them more comparable. According to the same threshold criteria but based on gene expression data, 116 and 133 genes that were differentially regulated by E2 stimulation were filtered in MCF7 and OHT cell lines, respectively. Based on the corresponding PolII data we found 292 and 386 genes that were differentially regulated by E2 stimulation in MCF7 and OHT cell lines, respectively. And there were only a few genes overlap between differentially expressed genes and differentially PolII binding quantity genes, 6 in MCF7 and 8 in OHT. The difference in the number of differentially regulated genes implied that PolII binding quantity data are able to provide more regulatory details compared with gene expression data (Table 1). At P < 0.01, the numbers of predicted regulatory TFs based on PolII data in MCF7 and OHT cells were 41 and 48, respectively, which were significantly more than the corresponding values of 20 and 30 obtained from gene expression data (Table 1). To verify the accuracy of our predictions, we also determined the expression levels of genes that encode regulatory TFs in MCF7 and OHT cells. Among all of 460 TFs investigated, we found numbers of TFs expressed in MCF7 and OHT cells were 186 and 184, respectively. Specifically, 22 of 41 predicted regulatory TFs were expressed in MCF7 cells, and only 25 of 48 were expressed in OHT cells based on PolII data. In results obtained from gene expression data, only 9 of 20 predicted regulatory TFs were expressed in MCF7 cells, and only 8 of 30 were expressed in OHT cells (Table 1). Overall, predictions of regulatory TFs based on PolII data provided greater quantity and better accuracy compared with those based on gene expression data. Finally, we analyzed differentially regulated genes based on gene expression and PolII data using IPA (Ingenuity® Systems, http://www.ingenuity.com) to compare the results of enrichment analysis. Overall, not only in their associated diseases and disorders but also in the molecular and cellular functions of MCF7 and OHT, more molecules were found using PolII data than gene expression data at a significance level of P <0.05. Three categories of diseases and disorders in MCF7 results were found using both sets of data. Molecules with greater enrichment in PolII data than in gene expression data more frequently belonged to the categories of developmental disorder and cancer and less frequently belonged to the categories of skeletal and muscular disorders (Figure 2A). In OHT, molecules found to be more highly enriched in PolII data than in gene expression data belonged to the categories of cancer and endocrine system disorders. These categories were closely associated with breast cancer (Figure 2B). An analysis of cellular functions using PolII data also revealed that more molecules were enriched in the categories of cell cycle, cellular function and maintenance, and cell death and survival (Figure 3). These entries demonstrated that, regarding the specific function of regulation in breast cancer cells, data based on the quantity of PolII binding at promoter regions could provide better specifics for prediction. We also manually verified these important regulatory TFs in breast cancer cells in literature, such as AP-1, AP-2, C/EBP, E2Fs, ER, FoxA1, Oct-1, NF-κB and others. All of these findings confirmed that predictions based on PolII data could provide more recognized important regulatory TFs compared with gene expression data.

**Table 1:** A summary of differentially regulated genes, regulatory TFs and verified TFs identified from MCF7 and OHT cells.

	MCF7			OHT		
	
	*Based on gene expression data*	*Based on PolII binding quantity data*	*Results compared between PolII data and gene expression data*	*Based on gene expression data*	*Based on PolII binding quantity data*	*Results compared between PolII data and gene expression data*
**Differentially regulated genes**	116	292	+ 176	133	386	+ 253
**Identified regulatory TFs**	20	41	+ 21	30	48	+ 18
**Verified TFs in prediction**	9	22	+ 13	8	25	+ 17
**All verified TFs**	186	186	-----	184	184	-----

### Comparison of predictions based on PolII binding quantity in MCF7 and OHT cells

**Figure 4:**
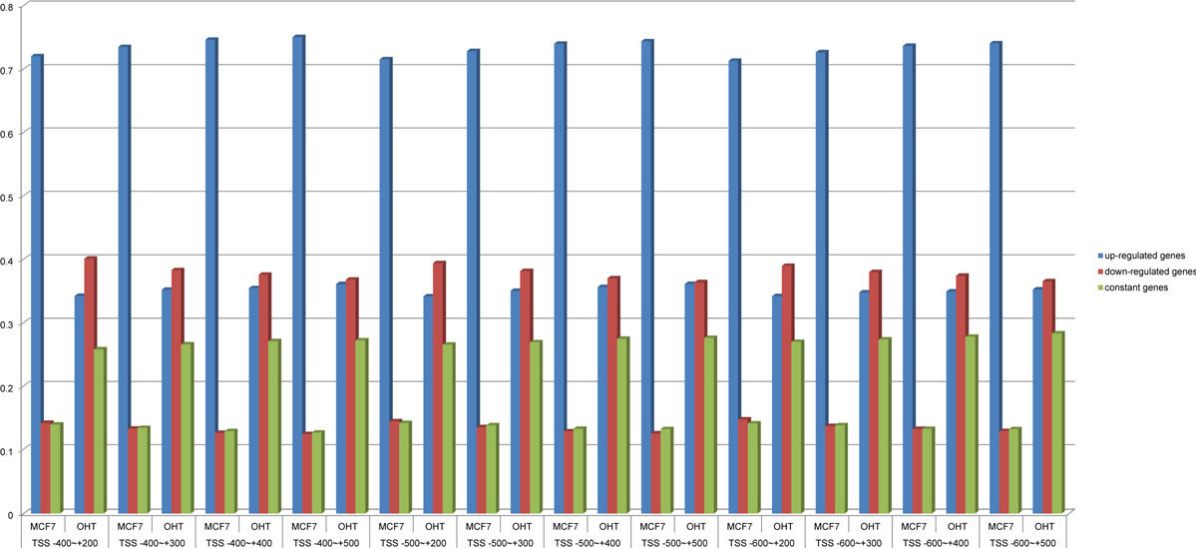
**Significant differences were found in PolII binding patterns between MCF7 and OHT cell lines**. We defined the variance of PolII binding quantity as follows: no more than +-2.5% of the average of PolII binding quantity before and after E2 stimulation indicated that PolII binding mode was constant; more than +2.5% of the variance in PolII binding quantity indicated that the gene was up-regulated, and less than -2.5% of the variance indicated that it was down-regulated. The percentages of up-regulated, down-regulated and constant genes identified based on PolII binding quantities in all 12 areas around the 34,055 TSSs of genes in MCF7 and OHT cells when stimulated by E2 appeared to be significantly different. This finding implies great difference between the transcriptional regulation modes of two cell lines.

The specific biological mechanism of endocrine resistance in breast cancer therapy is very complex. We studied the changes of transcriptional regulation level in estrogen-dependent MCF7 and estrogen-independent OHT cell lines under E2 stimulation. We compared the differential variance in PolII binding quantity of 34,055 genes at approximately TSS -400 bp ~ +200 bp to -600 bp ~ +500 bp under E2 stimulation in MCF7 and OHT cells (Figure 4). We defined the variance of PolII binding quantity in following manner: no more than ±2.5% of the average of PolII binding quantity before and after E2 stimulation indicated PolII binding mode was constant; more than +2.5% of the variance in PolII binding quantity indicated gene was up-regulated; and less than -2.5% of the variance indicated gene was down-regulated. As demonstrated in Figure 3, after stimulation with E2, the number of up-regulated genes was far greater than down-regulated and constant genes in the MCF7 cells. However, the number of down-regulated genes was slightly more than up-regulated and constant genes in OHT cells under E2 stimulation. These significant differences in PolII binding modes between MCF7 and OHT cells were also mentioned in literature [16,27,28], indicating a very large difference in the transcriptional regulation mode, which could be one of the potential causes of endocrine resistance in breast cancer cells.

Based on PolII data and following our two-step workflow, we predicted 41 and 48 regulatory TFs in MCF7 and OHT cells, respectively (Table 2). There were some regulatory TFs discovered in both two cells, such as AP-2, BSAP, E2F, E2F-1, E2F-4: DP-2, ER, HIF-1, MAZR, TCF11: MafG, YY1 and ZID. AP-2 is involved in cell proliferation, differentiation, apoptosis and carcinogenesis. Although the family of AP-2 promotes the growth and differentiation of breast cancer [29], but continued expression of AP-2 has been correlated with a better prognosis [30]. BSAP is considered a key TF in the directed differentiation of B cells [31] and is considered to inhibit immunoglobulin activity [32]. The E2F family involves in cell cycle regulation and controls the nuclear proto-oncogene c-Myc [33]. The family member E2F-1 plays a crucial role during the G1 phase/S phase transformation [34]. The binding of estrogen to ER induces the activation of receptor and enhances ER-driven transcription to regulate proliferation and differentiation of breast cancer. Hypoxia is a common phenomenon of solid tumors, and HIF-1 can induce a series of genes which associate with angiogenesis and anaerobic metabolism and lead to tumor cells adapt to the hypoxic environment [35]. HIF-1 also plays an important role in the regulation of tumor cell apoptosis, invasion, spread, and radiotherapy or chemotherapy resistance [36]. YY1 (TF Yin Yang 1) not only controls DNA damage, DNA recombination, DNA repair and differentiation but also many divergent cellular processes, including cell proliferation and apoptosis, depending on targeted genes, cofactors and cellular environment [37,38]. YY1 is also considered in association with development of a malignant phenotype in some human cancers, which could indicate metastasis or survival [39,40]. Through verifying the GO annotation and related literature manually, we found the shared regulatory TFs between MCF7 and OHT cells are mainly involved in proliferation, differentiation and apoptosis. From a functional perspective, compared with the shared regulatory TFs in both cell lines, some regulatory TFs that are specific to OHT cells could play more critical roles in endocrine resistance of breast cancer. For example, Oct-1 can bind to the *ESR1 *promoter (the gene that encodes ERalpha) to promote transcription [41]. This response can be regarded as an adaptive response to estrogen inhibition by breast cancer cells by generating a large amount of ER to counteract the inhibitory effect of TMA. AP-1 (activator protein 1) is a fundamental factor for ERα-mediated transcription. By binding to regulatory elements in the E2F1 promoter, AP-1 and the ER cooperate in regulating E2F1 gene expression [42]. FoxA1 exhibits the greatest extent of mitotic chromosome binding [43] and is essential for AHR-dependent (aryl hydrocarbon receptor) regulation of cyclin G2 [44]. C/EBPs are a highly conserved family of leucine zipper-type (bZIP) DNA-binding proteins and have been implicated in cellular proliferation, terminal differentiation and apoptosis in a variety of tissues, including the mammary gland [45]. In human breast cancer, GATA1 as a negative transcriptional regulator binds the *Peroxiredoxin 5 *(*Prx5*) gene, and overexpression of *Prx5 *in mammary tissue is associated with the inhibition of apoptosis and poor prognosis [46]. Some studies have shown suppression of nuclear factor NF-kappaB (NF-κB) activation can suppress tumor growth in breast cancer [47].

**Table 2:** Summary of regulatory TFs identified from MCF7 and OHT cells with E2 treatment.

Regulatory TFs in MCF7	Regulatory TFs in OHT
AP-2, AP-2gamma, BSAP, CACCC-binding factor,	Oct-1, AHRHIF, AP-1, AP-2,
CDP CR1, c-Myb, c-Myc:Max, Crx,	AR, ATF4, ATF6, BSAP,
E12, E2F, E2F-1, E2F-1:DP-1,	C/EBP, C/EBPdelta, Cdc5, E2F,
E2F-4:DP-2, E47, EGR, Egr-2,	E2F-1, E2F-4:DP-2, En-1, ER,
ER, ETS, GATA-2, GATA-3,	Evi-1, FoxA1, FOXO1, FOXO4,
HEB, HIF-1, Hmx3, IRF-7,	GATA-1, Hand1:E47, HeliosA, HFH-3,
LXR, LXR/PXR/CAR/COUP/RAR, MAZR, MTF-1,	HIF-1, Ik-3, IPF1, IRF,
MYB, Ncx, Nrf-1, NRSF,	LEF1TCF1, MAZR, MEIS1A:HOXA9, Msx-1,
p53, PAX6, Pax-8, SMAD-4,	NF-kappaB, NF-Y, Nrf2, PPARalpha:RXR-alpha,
TCF11:MafG, USF, YY1, ZF5,	RORalpha2, RP58, SF-1, SRF,
ZID	TATA, TCF11:MafG, TEF, Tel-2,
	TFIIA, TGIF, YY1, ZID

From above analysis, we find specific regulation by TFs maintained OHT cells with molecular interactions and signaling pathways are different from those of surviving MCF7 cells. These molecular interactions and signaling pathways are often not mutually exclusive and instead have complex interactions and cross-talk with one another. A mutual substitution effect between these molecular interactions and signaling pathways may be the primary cause of endocrine resistance in breast cancer.

### Different interactive modes of TFs between MCF7 and OHT cells

**Figure 5:**
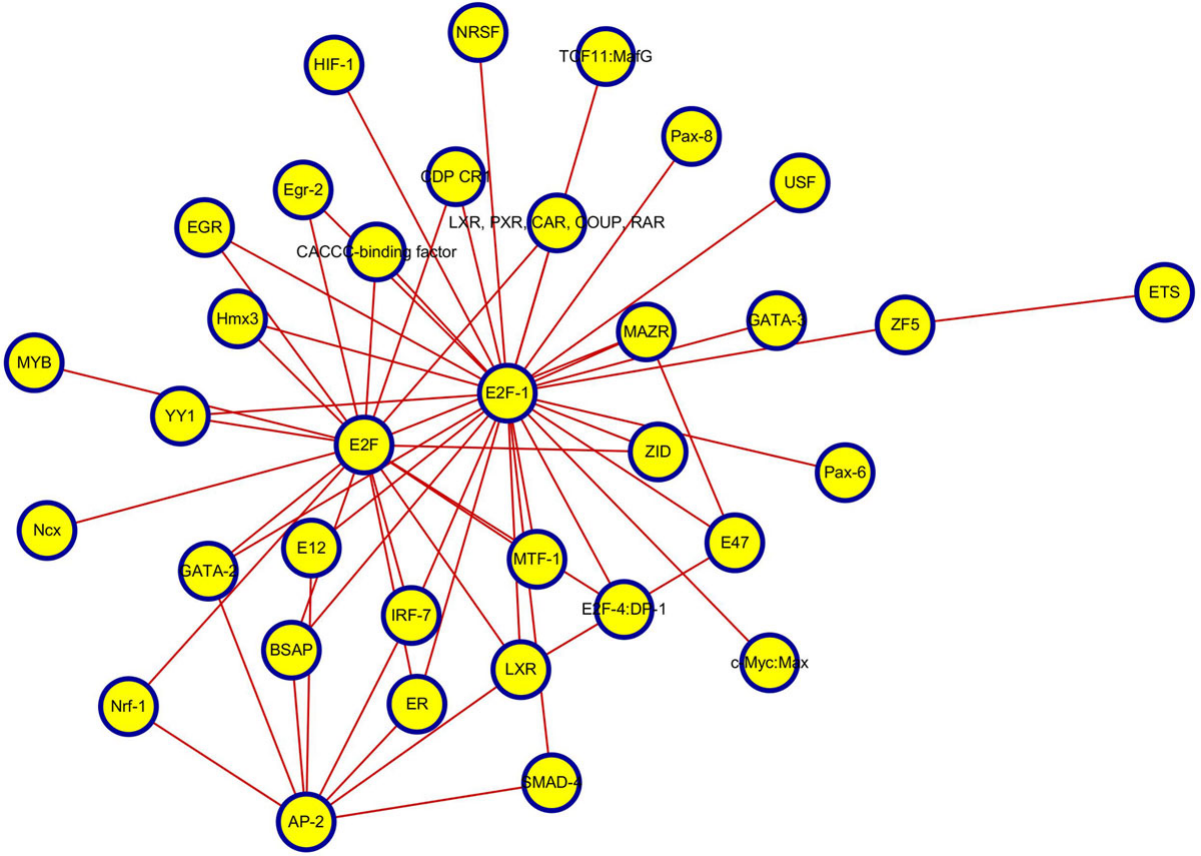
**TF regulatory interaction network in MCF7 cells**. We proposed the SIS as a measure to compare the significance of the interaction between two TFs and constructed the TF regulatory interaction network based on the SIS scores between TFs in MCF7 cell line. The nodes represent regulatory TFs involved in interactions, and the edges represent significant interactions between TFs. The hub-TFs in MCF7 cells were E2F, E2F-1 and AP-2.

**Figure 6:**
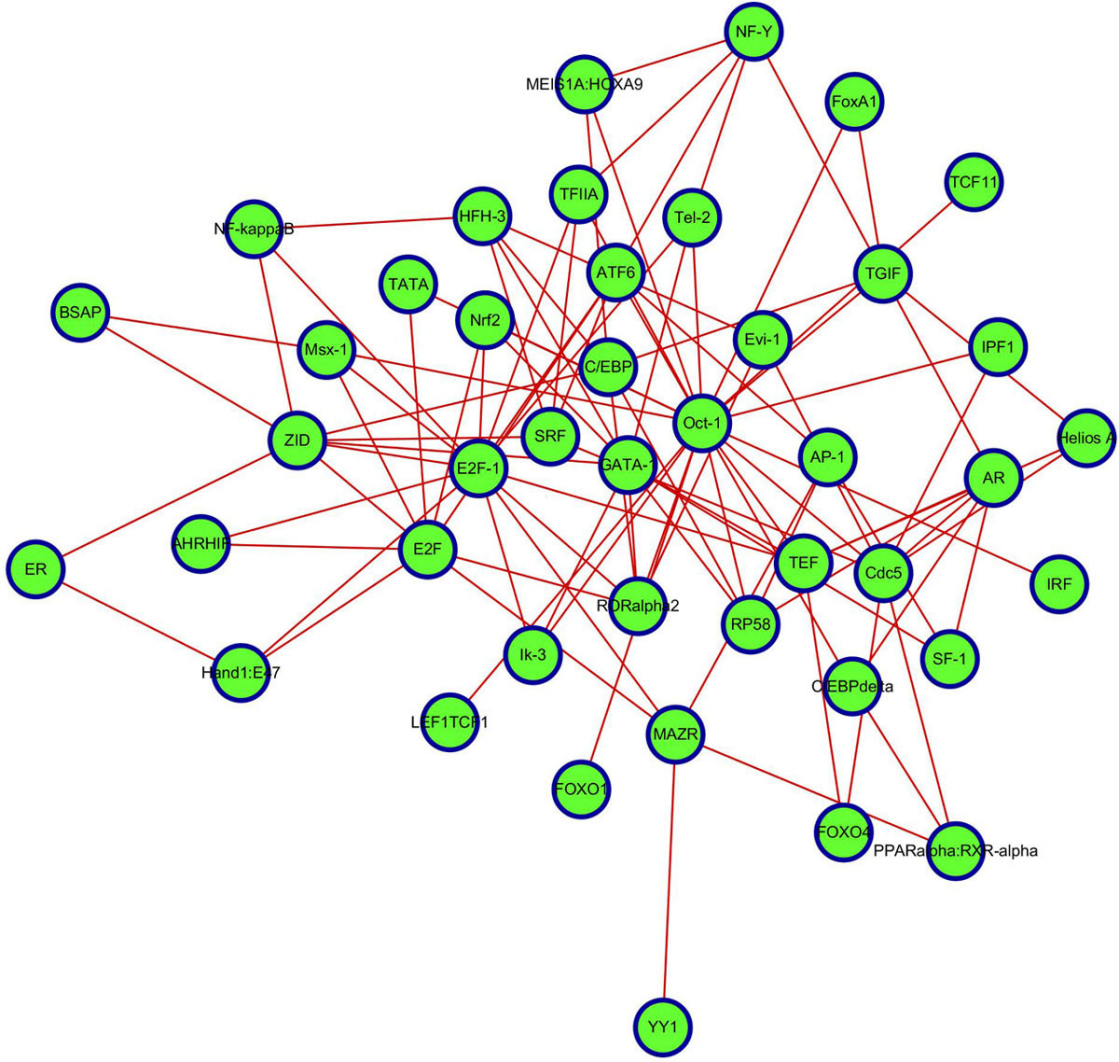
**TF regulatory interaction network in OHT cells**. We proposed the SIS as a measure for comparing the significance of the interaction between two TFs and constructed the TF regulatory interaction network based on the SIS scores between TFs in OHT cell line. The nodes represent regulatory TFs involved in interactions, and the edges represent significant interactions between TFs. Hub-TFs in OHT cells included C/EBP and Oct-1, in addition to E2F and E2F-1.

Collaboration through interactions between one another is the main manner in which TFs play a role in intracellular regulation. To further understand the differences in the TF regulatory mode between MCF7 and OHT cells under E2 stimulation, we constructed TF regulatory interaction networks in two cell lines using SIS scoring and setting the mean + variance as the threshold of significance, as shown in Figures 5 and 6 below.

Difference of regulatory patterns was apparent from TF regulatory interaction networks. Obvious hub-TFs in MCF7 cells were E2F, E2F-1 and AP-2; but in OHT were C/EBP and Oct-1, in addition to E2F and E2F-1. In MCF7 cells, E2F family was involved in cell cycle regulation and tumor promotion through the regulation of the proto-oncogene c-Myc. However, E2F in OHT cells could play another role, such as in CDK4/Rb/E2F transcriptional axis in the hormone-independent growth of breast cancer cells [25]. This arrangement implies that E2Fs assist in antiestrogen resistance in estrogen-independent breast cancers. In general, *BRCA1 *(breast-cancer-associated gene 1) interacting with Oct-1 tends to cause breast cancer cells that show higher levels of chromosomal abnormalities [48]. Additionally, Oct-1 can bind to the promoter of *ESR1 *(the gene that encodes ERalpha) to promote ER transcription. In breast cancer cells, TMA binds to the AF2 domain of the ER to block its activity; as a result, promoting Oct-1 in OHT can be regarded as an adaptive response to estrogen inhibition that estrogen-independent tumor cells undergo by generating a large number of ERs to counteract the inhibitory effect of TMA. Down-regulating and inhibiting C/EBP proteins could cause breast cancer cells to evade apoptosis and grow in an uncontrolled fashion [49].

We investigated the C/EBP gene expression found before and after E2 stimulation in two cell lines. C/EBP expression was not significantly changed in MCF7 cells, but in OHT cells, expression of C/EBP was 1788.25, which is far less than the 2323.75 in MCF7 cells; this finding implies that the change in the regulatory pattern of C/EBP could be associated with estrogen resistance. The major regulatory TFs and significant interactions between TFs that were revealed in TF regulatory interaction network further our understanding of the mechanism of TAM resistance in breast cancer.

### Identified important TFs and regulatory TFs with low signal intensity

**Figure 7:**
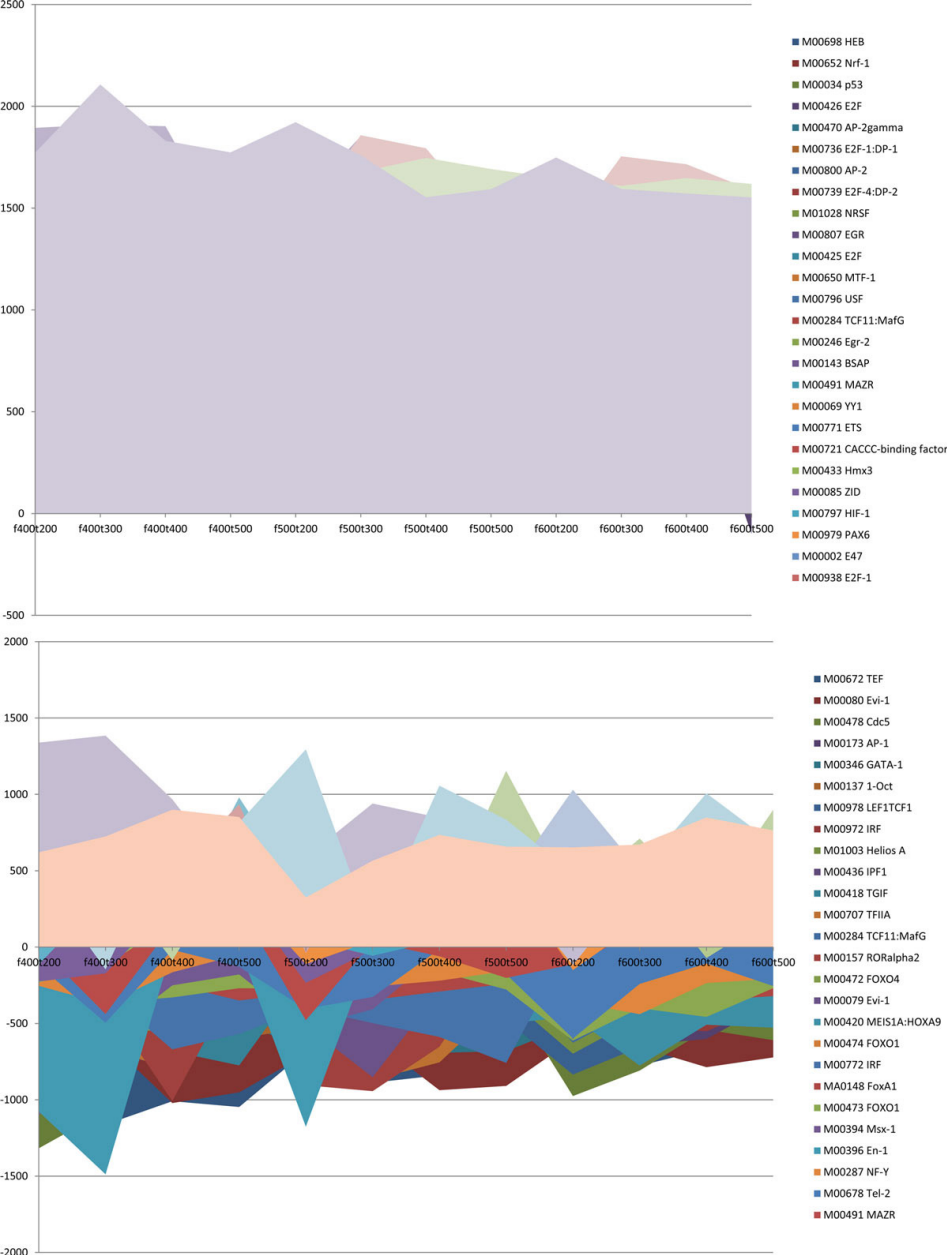
**Comparisons of TF regulatory ability between each interval in MCF7 and OHT cells**. The horizontal axis represents different intervals, and the vertical axis represents regression coefficient, which reflects the signal intensities of regulatory TFs. The regulatory intensities of TFs such as c-Myc; GATA-2 in MCF7; and Evi-1 and YY1 in OHT were so large over all of the regions that the signal fully submerged those smaller regulatory signal intensities of other TFs.

**Figure 8:**
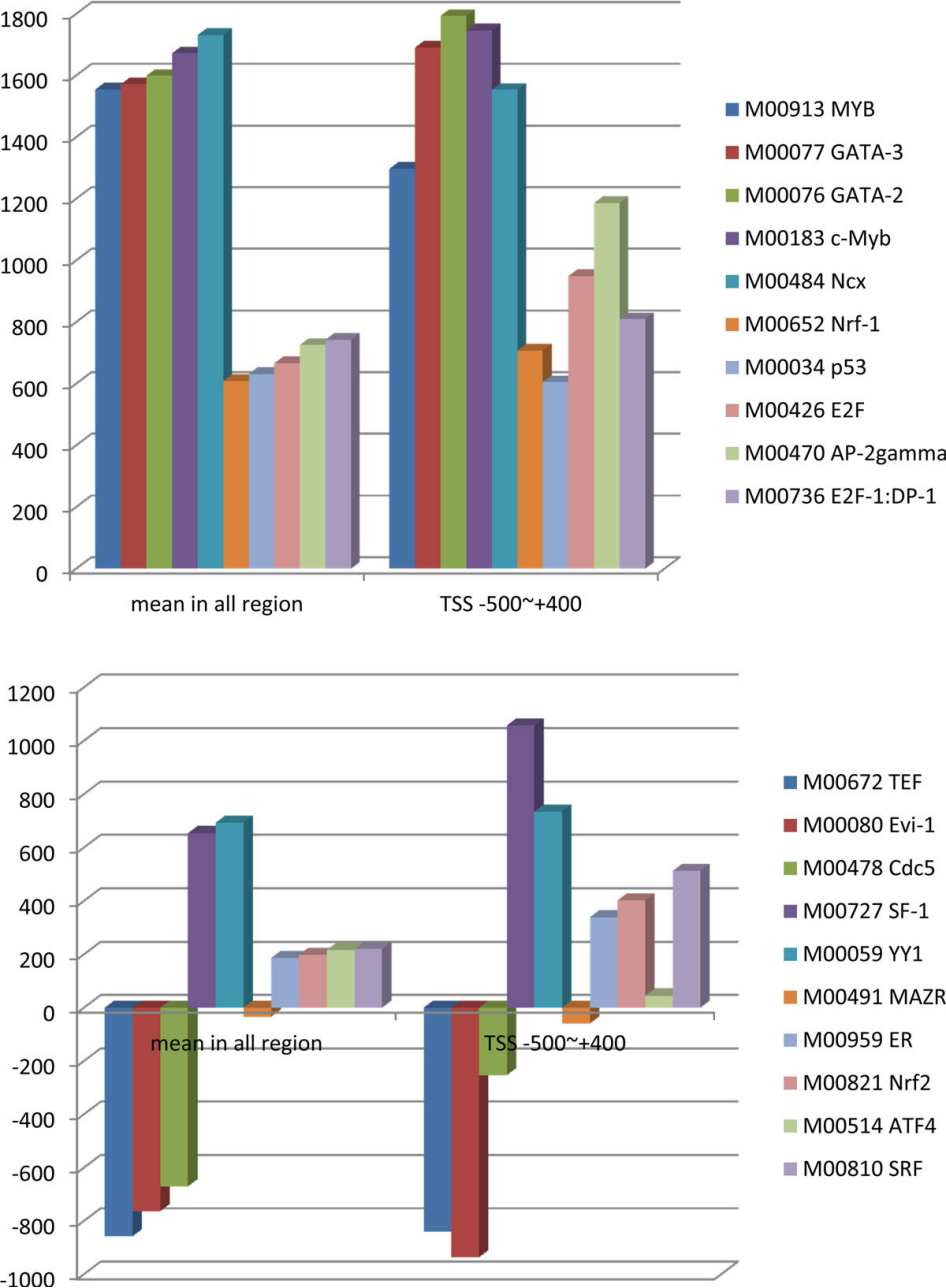
**Comparisons of the top five TFs with the maximum and minimum regulatory intensities in MCF7 and OHT cells**. The horizontal axis represents different intervals, and the vertical axis represents regression coefficient, which reflects the signal intensities of the regulatory TFs. The top five TFs with the maximum and minimum regulatory intensities were selected from MCF7 and OHT cells, and their average intensities were compared with their regulatory intensity in TSS -500 bp ~ +400 bp region. Over all of the regions compared, regulatory intensities of the top five strongest regulatory TFs were much greater than those of the weakest five TFs. However, in a specific area, such as TSS -500 bp ~ +400 bp interval, regulatory intensity of AP-2gamma, which came from the smaller regulatory intensity groups, was almost as strong as that of MYB in the maximum regulatory intensity group. A similar phenomenon was founded in OHT cells, too.

The regulatory capacity of different TFs varies greatly in cells. The traditional linear prediction method tends to model regulatory elements in a fixed area based on gene expression, but it failed to predict those important TFs with low regulation intensity. We proposed a regression model based on the sub-regional PolII binding quantity, which reflects the regulatory intensity of TFs in a specific range, to find TFs that were important but had low signal intensity. As shown in Figure 7, for some TFs (such as c-Myc; GATA-2 in MCF7; and Evi-1 and YY1 in OHT), their regulatory signal intensity was so large over all of the regions that the signal fully submerged the smaller regulatory signal intensities of other TFs. We selected the top 5 TFs with the maximum and minimum regulatory intensities from MCF7 and OHT and compared their average intensity with regulatory intensity in regions TSS -500 bp ~ +400 bp, as shown in Figure 8. Over all of regions, regulatory intensities of the top 5 strongest regulatory TFs were much larger than those of the weakest 5 TFs. However, in TSS -500 bp ~ +400 bp interval, we found that the regulatory intensity of AP-2gamma, which was one of the smaller regulatory intensity groups, was almost as strong as that of MYB in the maximum regulatory intensity group. We also found a similar phenomenon in OHT. Over all of regions, the regulatory intensity of weaker TFs would be significantly enhanced in the vicinity of its true binding sites, which would enable our method to distinguish important regulatory TFs with weaker signal intensities from other TFs that have strong regulatory signals.

### Regulatory role of different motifs of the same TF

To improve prediction accuracy, our model used all of known motifs of each TF for prediction. We compared the regulatory intensities of predicted motifs of the same TF in all of different areas and found some interesting situations.

**Figure 9:**
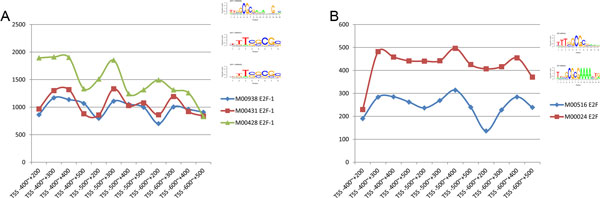
**Motifs of the same TF in all of different areas showed similar patterns of regulation**. The horizontal axis represents different intervals, and the vertical axis represents regression coefficient, which reflects the signal intensities of the regulatory TFs. The order of logos corresponds to the respective motif. Although different motifs of E2F-1 in MCF7 cells and E2F in OHT cells varied in terms of their quantities of regulatory ability, their regulatory patterns were very similar.

**Figure 10:**
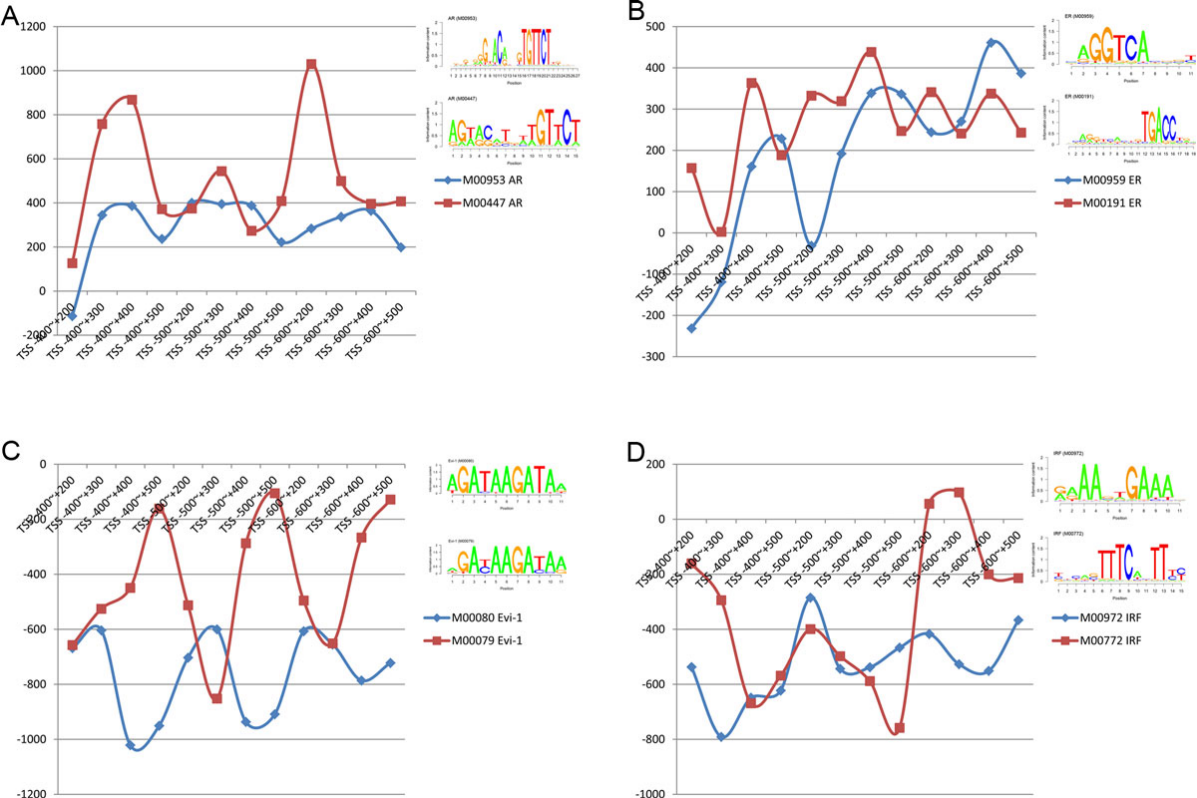
**Motifs of the same TF in all of different areas showed different patterns of regulation**. The horizontal axis represents different intervals, and the vertical axis represents regression coefficient, which reflects the signal intensities of the regulatory TFs. The order of logos corresponds to the respective motif. In OHT cells, motifs of AR in both TSS -400 bp ~ +300 bp and TSS -600 bp ~ +200 bp were different. The ER and IRF motifs even exhibited a regulation change in direction. In particular, the regulatory patterns of Evi-1 over the entire range were nearly opposite while their motifs were very similar.

TF motifs in same cells of same environment usually play a similar role in regulation. Indeed, we observed this situation in both MCF7 and OHT. As shown in Figure 9, although different motifs of E2F-1 in MCF7 and E2F in OHT cell lines varied in terms of the quantity of regulatory ability, their regulatory patterns were very similar. However, we found motifs of the same TF sometimes showed different patterns of regulation (see Figure 10). In OHT, for example, motifs of AR in both TSS -400 bp ~ +300 bp and TSS -600 bp ~ +200 bp nearby were different and the ER and IRF motifs even had a regulation change in the direction that emerged. In particular, the regulatory patterns of Evi-1 over the entire range studied were nearly opposite but their motifs were very similar.

These identified similar or different regulatory patterns of motifs confirmed that the resolution of our model for predicting TFs was very high. The motifs of the same TF have different regulatory patterns, which also indicated that these TFs could have a relatively complex regulatory manner and could play different roles in regulation.

## Discussion

Estrogen action through its cognate receptor, the ER, is the most important mechanism in breast cancer. The principal action modes of the ER include classical genomic, non-classical genomic and non-genomic modes. In classical genomic action mode, ER directly binds estrogen response elements (EREs) in promoter regions of regulated genes and recruits co-activators or co-repressors to modulate gene transcription [5]. In non-classical genomic action mode, ER does not require EREs but instead mediates transcription by the interactions of ERa with other proteins, including AP-1(Fos/Jun) [50,51], AP-2 [52,53], C/EBP [49,54], E2Fs [55-57], FoxA1 [58,59], Oct-1 [55,60], NF-κB [51,61] and others [62-64]. In addition, non-genomic action of estrogen often includes ligand binding to the ER at the plasma membrane and follows the activation of signaling pathways. It has been demonstrated that several signaling kinases interacted with and were activated by the ER, including IGF-1R (insulin-like growth factor-1 receptor), Src, PI3K (phosphatidylinositol 3-kinase), MAPK (mitogen-activated protein kinase), protein kinases A and C and calcium pathways [65], EGFR (epidermal growth factor receptor) and ErbB-2, with non-genomic effects of the ER [66,67]. Furthermore, genomic and non-genomic mechanisms of action of ER are not mutually exclusive, but many interactions exist between these two modes [4].

For estrogen resistance, TAM primarily inhibits ER action through direct competitive binding to the AF-2 domain of ER; TAM can also disrupt protein-protein interactions between ER and its binding partners and thus inhibit the activity of ER in supporting breast cancer sustainable survival [68,69]. Therefore, the expression level of ER was significantly decreased in OHT compared with MCF7 cells, which primarily causes an inherent resistance to endocrine therapies [70]; this phenomenon was also found in our experiments. Despite the supersensitivity and hypersensitivity to estrogen could compensate for reduction of estrogen in estrogen-deficient cellular environment, similar to OHT and as shown in [71,72], it is more likely that there are different mechanisms from the ER involved alternative growth regulatory pathways in antiestrogen resistance to support tumour cells' sustainable survival. According to our predictions, both NF-κB and AP-1, which are ER-associated TFs and co-activators and usually promote the ER effect through protein-protein interactions, could be found in specific regulatory TFs of OHT cells. Previous studies showed phosphorylation and over-expression of these co-regulators can cause an increase in ER-mediated transcription associated with endocrine resistance [73]. On the other hand, under TAM treatment, p38γ/MAPK was selectively activated, c-Jun transcription was stimulated, and ER signaling was switched from the classical ERE binding to an interaction with AP-1. Both of these led to increased hormone sensitivity and promoted breast cancer growth [74]. TFs participate in both molecular interactions and signaling pathways may be primarily responsible for antiestrogen resistance in breast cancer.

In redox environment, ROS (reactive oxygen species) can lead to breast cancer cells proliferation either through AP-1 and NF-κB binding sites or through the MAPK/AP-1 and ROS/NF-κB pathways [75]. Oxidative stress is implicated in increased AP-1 DNA binding with c-JUN NH2 terminal kinase activity in TAM-resistant breast cancer [63]. Furthermore, some experimental studies reported the crosstalk between ER, especially mitogen-activated protein kinase MAPK/ERK and phosphatidylinositol 3-kinase PI3K/AKT signaling pathways along with signaling cascade up-regulation, which leads to the activation of AP-1, NF-κB and HIF-1, could be common in production of resistance to all forms of endocrine therapy [76]. Active NF-κB induced neoplastic transformation of mammary cells [77], and inhibition of NF-κB in breast cancer cells can induce spontaneous apoptosis [78]. Regulation of TFs through both protein-protein interactions and signaling pathways to adapt to different cellular environments could be one of resistant mechanism in endocrine therapy [79].

## Conclusions

Breast cancer that is resistant to antiestrogen therapy and relapse has been one of the most difficult problems in clinical treatment. Although resistance to antiestrogen in breast cancer has been extensively studied, its mechanism has not yet been completely elucidated. In this paper, we studied TF regulatory roles in resistance to antiestrogen therapy in breast cancer through our two-step workflow. Large differences in regulatory TF groups were found between estrogen-dependent MCF7 and estrogen-independent OHT cells. The mutual regulatory TFs found in both MCF7 and OHT cells were primarily involved in proliferation, differentiation and apoptosis. We found some regulatory TFs, such as AP-1, C/EBP, FoxA1, GATA1, Oct-1 and NF-κB, maintained OHT cells through molecular interactions or signaling pathways that were different from the surviving MCF7 cells. Regulation of TFs such as AP-1 and NF-κB through both protein-protein interactions and signaling pathways to adapt to different cellular environments could be one of resistant mechanism in endocrine therapy.

Different action modes in the two types of cells were compared, and the TF regulatory interaction networks of MCF7 and OHT were further constructed. Obvious hub-TFs in MCF7 cells were E2F, E2F-1 and AP-2; however, additional visible hub-TFs in OHT cells included C/EBP, Oct-1 outside E2F and E2F-1. Major regulatory TFs and the significant interactions between TFs that were revealed in TF regulatory interaction networks further our understanding of the mechanism of TAM-resistance in breast cancer, and these regulatory TFs and their interactions could also provide new proposed targets for treatment.

We also compared predictions obtained from gene expression data and PolII binding quantity data; based on the same analysis workflow, the results showed that PolII binding quantity data were able to provide better sensitivity and specificity for TF regulation prediction.

The traditional linear prediction method model gene expression and regulatory elements in a fixed area, but this approach failed to predict TFs that were important but had low regulation intensities because their regulatory signal intensities was overwhelmed by signal intensities of strong regulatory TFs. We proposed a workflow building regression model based on PolII binding quantity which reflects regulatory intensities of TFs in a specific range in a manner. Our workflow could detect those important regulatory TFs with weaker signal intensities.

TF motifs in same cells and same environment are usually expected to play similar roles in regulation. We compared the regulatory intensities of these motifs of the same TFs in all of the different areas and found that some played similar regulatory roles in both MCF7 and OHT. But motifs of the same TFs sometimes showed different regulatory patterns. These identified regulatory patterns not only demonstrated the high resolution of our model in prediction of TFs but also implied these TFs have a complex regulatory manner and play different roles in regulation depending on cellular environment or co-factors.

Focusing on the level of transcription regulation allows good predictive accuracy in our analysis workflow. We also believe that our flexible workflow can be further integrated into an existing analytical framework of epigenetic modification or post-transcriptional regulation, which can be used to obtain more meaningful results.

## List of abbreviations

TAM: Tamoxifen; E2: 17β-estradiol; TFs: transcription factors; PolII: RNA polymerase II; ER: estrogen receptor; CNV: copy number variation; EREs: estrogen response elements; LOWESS: locally weighted scatterplot smoothing; ChIP: Chromatin immunoprecipitation; TMRs: the total number of mapping reads; TFBSs: transcription factor binding sites; PWMs: position weight matrices; TSS: transcription start site

## Competing interests

The authors declare that they have no competing interests.

## Authors' contributions

DZ and GW contributed to the design of the study. DZ performed the study, conducted initial data analysis and wrote the manuscript. YW conceived, designed, and supervised the study. All authors read and approved the final manuscript.

## Declarations

The publication costs for this article were funded by China National 863 High-Tech Program (2012AA020404, 2012AA02A602, 2012AA02A601) and National Natural Science Foundation of China (61371179, 61173085).

This article has been published as part of *BMC Bioinformatics *Volume 15 Supplement 2, 2014: Selected articles from the Twelfth Asia Pacific Bioinformatics Conference (APBC 2014): Bioinformatics. The full contents of the supplement are available online at http://www.biomedcentral.com/bmcbioinformatics/supplements/15/S2.
